# Urban and rural prevalence of diabetes and pre-diabetes and risk factors associated with diabetes in Tanzania and Uganda

**DOI:** 10.3402/gha.v9.31440

**Published:** 2016-05-23

**Authors:** Faraja S. Chiwanga, Marina A. Njelekela, Megan B. Diamond, Francis Bajunirwe, David Guwatudde, Joan Nankya-Mutyoba, Robert Kalyesubula, Clement Adebamowo, IkeOluwapo Ajayi, Todd G. Reid, Jimmy Volmink, Carien Laurence, Hans-Olov Adami, Michelle D. Holmes, Shona Dalal

**Affiliations:** 1 Endocrinology and Diabetes Unit, Department of Internal Medicine, Muhimbili National Hospital, Dar es Salaam, Tanzania; 2 Department of Physiology, Faculty of Medicine, Muhimbili University of Health and Allied Sciences, Dar es Salaam, Tanzania; 3 Department of Epidemiology, Harvard T.H. Chan School of Public Health, Boston, MA, USA; 4 Department of Community Health, Mbarara University of Science and Technology, Mbarara, Uganda; 5 Department of Epidemiology and Biostatistics, School of Public Health, Makerere University College of Health Sciences, Kampala, Uganda; 6 Institute of Human Virology, Abuja, Nigeria; 7 School of Medicine Greenbaum Cancer Center and Institute of Human Virology, University of Maryland, Baltimore, MD, USA; 8 Department of Epidemiology and Medical Statistics, Faculty of Public Health, College of Medicine, University of Ibadan, Ibadan, Nigeria; 9 The South African Cochrane Centre, South African Medical Research Council, Cape Town, South Africa; 10 Centre for Evidence-based Health Care, Faculty of Medicine and Health Sciences, Stellenbosch University, Cape Town, South Africa; 11 Department of Medical Epidemiology and Biostatistics, Karolinska Institute, Stockholm, Sweden; 12 Channing Division of Network Medicine, Department of Medicine, Brigham and Women's Hospital and Harvard Medical School, Boston, MA, USA

**Keywords:** non-communicable, risk factors, underdiagnoses, sub-Saharan Africa, Tanzania, Uganda

## Abstract

**Background:**

The increase in prevalence of diabetes and pre-diabetes in sub-Saharan Africa underlines the importance of understanding its magnitude and causes in different population groups. We analyzed data from the Africa/Harvard Partnership for Cohort Research and Training (PaCT) studies to determine the prevalence of diabetes and pre-diabetes and risk factors associated with diabetes.

**Methodology:**

Participants were randomly selected from peri-urban (*n*=297) and rural (*n*=200) communities in Uganda, and teachers were recruited from schools (*n*=229) in urban Tanzania. We used a standardized questionnaire to collect socio-demographic and self-reported disease status including diabetes status. Blood glucose was also measured after participants fasted for 8 h. We used standard protocols for anthropometric and blood pressure measurement.

**Results:**

The overall prevalence of diabetes was 10.1% and was highest in rural Ugandan residents (16.1%) compared to teachers in Tanzania (8.3%) and peri-urban Ugandan residents (7.6%). The prevalence of pre-diabetes was 13.8%. The prevalence of self-reported diabetes was low across all sites, where 68% of participants with diabetes were not captured by self-report. In multivariable logistic regression analysis, family history (OR 2.5, 95% CI: 1.1, 5.6) and hypertension (OR 2.3, 95% CI: 1.1, 5.2) were significantly associated with diabetes.

**Conclusions:**

The prevalence of diabetes and pre-diabetes in Uganda and Tanzania is high, differs markedly between population groups, and remains undiagnosed in an alarmingly high proportion of individuals. These findings highlight the need for large-scale, prospective studies to accurately quantify the burden and identify effective intervention and treatment strategies across diverse African populations.

## Introduction

The International Diabetes Federation (IDF) estimates that 19.8 million people have diabetes in Africa where approximately 75% are still undiagnosed (1). Type 2 diabetes (T2D) contributes up to 90% of all cases of diabetes (2). The increase in diabetes prevalence in sub-Saharan Africa (SSA) has grown in parallel with the increase in obesity and other cardiovascular risk factors (3). Countries with the highest estimated numbers of persons with diabetes include Nigeria (3.9 million), South Africa (2.6 million), Ethiopia (1.9 million), and Tanzania (1.7 million) (1). Diabetes exerts a huge societal burden by reducing quality of life and life expectancy, as well as causing economic loss to individuals and nations (4).

Rapid urbanization, increasingly sedentary lifestyles, and unhealthy eating habits have contributed largely to the increased prevalence of diabetes, estimated to be 5.7% and expected to rise to 6% by 2035 (1). The prevalence of pre-diabetes, a transition stage with blood glucose levels higher than normal but not high enough to be diagnosed as diabetes, is currently at 8.3% and expected to rise to 9.3% by 2035. Therefore, interventions to control the epidemic of diabetes and hyperglycemia-related vascular complications should start at this early stage of its development (5).

The prevalence of diabetes in Tanzania and Uganda, two SSA countries with comparable socioeconomic status, is estimated at 7.8% (Tanzania) and 4.1% (Uganda), while impaired glucose tolerance is estimated at 9.1% in Tanzania and 6.6% in Uganda (1). The estimated number of undiagnosed patients is 469.3 per 1,000 and 1281.7 per 1,000 in Uganda and Tanzania, respectively (1). The health delivery service structure for Tanzania and Uganda is pyramidal with primary health care services at its base. Despite policy stating that primary care facilities should provide services for diabetes, studies have demonstrated that most dispensaries and health centers do not provide such services. Lack of guidelines, basic supplies, diagnostic tools, and training are the frequently cited reasons for the underutilization of primary health care in providing diabetes care (6, 7).

Although communicable diseases remain the most common causes of morbidity and mortality in low-income countries, the rapid increase in the prevalence of non-communicable diseases (NCDs) including diabetes creates a challenge for prevention and treatment. Data for diabetes in SSA are sparse and often from single-country studies. Lack of comprehensive studies on diabetes etiology and risk factors creates a knowledge gap (8). There is an urgent need to obtain local data in order to implement locally applicable preventive strategies.

We report on data from the Africa/HSPH Partnership for Cohort Research and Training (PaCT), an initiative that aims to conduct a large prospective study in South Africa, Tanzania, Uganda, and Nigeria to gain knowledge on risk factors for NCDs including diabetes. The aim of this analysis from pilot studies was to determine the prevalence of diabetes and pre-diabetes and its associated risk factors in Tanzania and Uganda.

## Materials and methods

### Recruitment

The PaCT study recruited participants from five African sites including teachers in Tanzania and South Africa, geographic residents from two sites in Uganda, and nurses in Nigeria. A full description of the study and participants has been described previously (9). We report here on data from a subset of participants from whom fasting blood glucose (FBG) was collected. These included 73.8% of the Tanzanian participants (169/229), 88.2% of the participants from peri-urban Uganda (262/297), and 77.5% of the participants from rural Uganda (155/200), which are described briefly below.

Participants from Tanzania were primary school teachers in Dar es Salaam, recruited from 18 randomly selected public schools in the Temeke District. Dar es Salaam is a major commercial city in Tanzania and is growing rapidly. According to the 2012 census, the population is 4.4 million people. Primary school teachers form the bulk of workforce in the public sector in Tanzania and have diverse social-economic backgrounds. At each selected school, the principal was contacted and in charge of distributing the questionnaire packets and instructions to the teachers. This packet included a written consent form and an appointment card for the Temeke District Hospital where physical measurements and a blood sample were provided. Teachers were asked to mail the questionnaires back in a pre-stamped envelope. In total, 229 teachers were enrolled.

Participants from Uganda were enrolled from two geographic regions: a peri-urban community in the Wakiso District, and a rural community in the Bushenyi District. Wakiso District is a peri-urban community, about 15 km from the capital Kampala. It is the most populated district in Uganda with a total population of two million and 59.2% of the population is within 5 km radius of a health unit (10, 11). Bushenyi, a predominantly rural district, is located in South Western Uganda and has a population of 250,000 of which 70% is within a walkable distance to a health facility (10, 12).

In Wakiso District, two parishes consisting of 13 villages were randomly selected for inclusion into the study. Participants were recruited in-person through house visits. Written consent was taken or read aloud if the participant was illiterate. In Bushenyi District, households were randomly selected from an enumerated list of all the households in each village. Trained research assistants recruited participants in-person through house visits. At both sites, interviews were done face-to-face and physical measurements and blood samples were taken on-site. In total, 297 people agreed to participate in peri-urban Uganda and 200 agreed to participate in rural Uganda.

### Data collection

The administered questionnaire was standardized across all sites and translated into kiSwahili in Tanzania and Luganda and Runyakitara in Uganda. The questionnaire relied on self-report of current medical conditions, family history of disease, and any treatment received. Main questions on diabetes included the following: Do you have a close family member(s) with diabetes or high blood sugar? Have you ever been told by a doctor or other health worker that you have high blood sugar or diabetes? Close family members included parents and first-degree relatives.

Blood glucose was measured in a fasting state (at least 8 h) at all three sites by finger prick using blood glucose meters that were calibrated for plasma glucose values. Diabetes mellitus was defined according to the WHO and IDF guidelines. Participants were categorized as having diabetes if their FBG levels were ≥7.0 mmol/l (126 mg/dl) *or* they self-reported having diabetes, and pre-diabetes if they had impaired fasting glucose defined as FBG of 6.1–6.9 mmol/l (110–125 mg/dl) with no self-reported history of diabetes (13, 14).

Standardized approaches for physical measurements including height, weight, abdominal circumference, and blood pressure were used at all three sites. These were recorded by health providers at Temeke District Hospital in Tanzania, and by trained study staff in Uganda. Physical measurements were used to determine body mass index (BMI). A BMI of 25–29.9 kg/m^2^ was classified as being overweight, a BMI of ≥30 kg/m^2^ as being obese and a BMI of <18.5 kg/m^2^ as underweight. Abdominal obesity was defined as having a waist circumference of >102 cm in males and >88 cm in females. Three blood pressure measurements were taken in sitting position at least 5 min apart using a digital blood pressure device. The average of the last two readings was used for analysis. Hypertension was defined as having a systolic blood pressure ≥140 mmHg or a diastolic blood pressure ≥90 mmHg or self-reporting hypertension.

### Ethical approval

Ethical approval was obtained through The Harvard School of Public Health Institutional Review Board; Makerere University School of Public Health Higher Degrees Research and Ethics Committee; Mbarara University of Science and Technology Research Ethics Committee; and the Uganda National Council of Science and Technology; and National Institute for Medical Research, Tanzania.

### Statistical analysis

Statistical analysis was performed using SPSS version 16 software. Frequencies and percentages were calculated. Chi square or Fisher's exact test was used to compare differences between groups. Variables with *p*<0.1 in univariate analysis were further analyzed with multivariable logistic regression to determine their independent association with diabetes. Differences between groups were considered significant if the p value was <0.05.

## Results

### Participant characteristics

Out of a total of 726 participants recruited in Tanzania and Uganda, 586 provided blood samples and had valid blood glucose readings. The characteristics of study participants are shown in Table 1. The distribution of participants by site was 26.5% from rural Uganda, 44.7% from peri-urban Uganda, and 28.8% from urban Tanzania. Across all sites, the majority of participants were females (61.4%) and the mean age was 36.8 (±13.4) years ranging from 18 to 80 years old. Uganda peri-urban participants were relatively younger (mean age 36.1 years), compared to rural Uganda (37.0 years) and Tanzania (40.0 years).

**Table 1:** Characteristics of the study population by sites

Characteristic	Total *n* (%)	Uganda rural *n* (%)	Uganda peri-urban *n* (%)	Tanzania *n* (%)
Overall	586 (100)	155 (26.5)	262 (44.7)	169 (28.8)
Sex				
Male	226 (38.6)	75 (48.4)	125 (47.7)	26 (15.4)
Female	360 (61.4)	80 (51.6)	137 (52.3)	143 (84.6)
Age groups				
18–29	144 (24.6)	36 (23.2)	91 (35.1)	16 (9.5)
30–39	170 (29.0)	57 (36.8)	51 (19.5)	62 (36.7)
40–49	112 (19.1)	35 (22.6)	35 (13.4)	42 (24.9)
≥ 50	94 (16.0)	19 (12.3)	37 (14.1)	38 (22.5)
Mean age (SD)	36.8 (13.4)	37.0 (10.6)	36.1 (15.4)	40.0 (9.3)
Smoking status				
Never smoked	510 (87.0)	113 (72.9)	233 (89.3)	164 (97.0)
Ever smoked	75 (12.8)	42 (27.1)	28 (10.7)	5 (3.0)
Body mass index				
Normal	251 (43.0)	76 (50.0)	145 (58.2)	31 (25.6)
Underweight	17 (2.9)	4 (2.6)	13 (5.2)	0 (0.0)
Overweight/obesity	253 (43.2)	72 (47.4)	91 (36.5)	90 (74.4)
Waist circumference				
Normal	282 (48.1)	123 (79.4)	113 (43.1)	46 (37.3)
Abdominal obesity^a^	257 (43.9)	32 (20.6)	149 (56.9)	76 (62.3)
Family history of diabetes^b^				
No	471 (80.7)	143 (92.3)	200 (76.6)	128 (76.2)
Yes	113 (19.3)	12 (7.7)	61 (23.4)	40 (23.8)
Hypertension^c^				
No	349 (59.6)	108 (83.1)	191 (73.7)	50 (37.6)
Yes	173 (29.5)	22 (16.9)	68 (26.3)	83 (62.4)

a Waist circumference>102 cm in males and >88 cm in females

b Parents and first-degree relatives

c Systolic BP of ≥140 or diastolic BP ≥ 90 or self-report.

### Prevalence of diabetes and pre-diabetes

Table 2 summarizes the prevalence of diabetes and pre-diabetes across all sites. Overall, 10.1% of participants had diabetes as defined by FBG or self-report and 13.8% had pre-diabetes. The prevalence of diabetes and pre-diabetes was higher in rural Uganda (16.1 and 26.5%, respectively) compared to other sites.

**Table 2:** Prevalence of diabetes and pre-diabetes by site

	Diabetes^a^ *n* (%)	Self-reported diabetes^b^ *n* (%)	Pre-diabetes^c^
Overall	59 (10.1)	22 (3.8)	81 (13.8)
Uganda rural residents	25 (16.1)	1 (0.6)	41 (26.5)
Uganda peri-urban residents	20 (7.6)	7 (2.7)	36 (13.7)
Tanzania urban teachers	14 (8.3)	14 (8.3)	4 (2.4)

a Fasting blood glucose (FBG) of ≥7.0 mmol/l or self-report

b Self-report only

c FBG of 6.1 mmol/l to 6.9 mmol/l and never been diagnosed with diabetes.

**Fig. 1:**
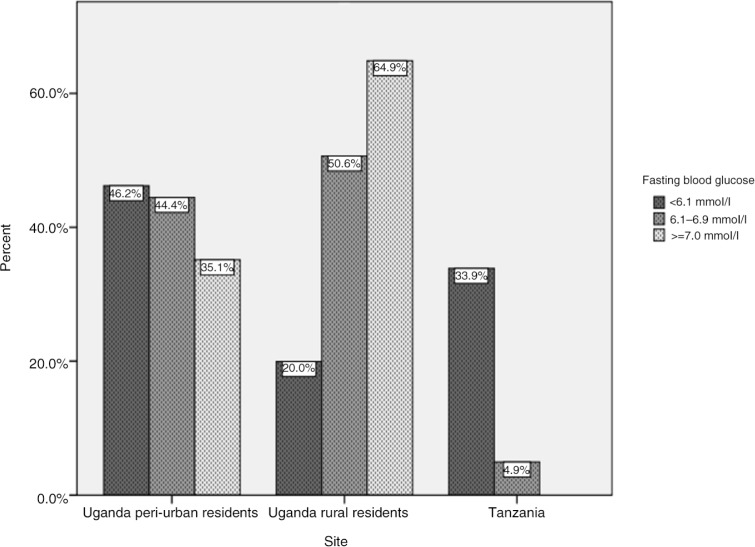
Fasting blood glucose among patients with no self-reported diabetes.

Diabetes prevalence versus self-reported diabetes status is presented in Table 2 and Fig. 1. The overall prevalence of self-reported diabetes was 3.8% (22/586), while the prevalence of diabetes, defined by having an FBG ≥7.0 mmol/l or self-reporting diabetes status, was 10.1%. Self-reported diabetes prevalence was lower compared to the calculated diabetes prevalence in peri-urban Uganda (2.7% vs 7.6%) and rural Uganda (0.6% vs 16.1%), while in Tanzania, all cases of diabetes were captured by self-report (8.3%). Underdiagnoses of diabetes were more pronounced in rural Uganda, where 95.6% of participants who had FBG≥7.0 mmol/l self-reported that they did not have diabetes. In peri-urban Uganda, 65.0% of participants who had FBG≥7.0 mmol/l self-reported that they did not have diabetes (Fig. 1). Overall, 68.2% of cases were not captured by self-report.

### Risk factors for diabetes

Association of selected risk factors for diabetes is presented in Table 3. Persons from rural Uganda were twice as likely to develop diabetes compared to those in other sites (16.1% versus 8.3% versus 7.6%). Factors that were statistically significantly associated with diabetes in bivariate analysis were 50 years old or older (OR 3.3, 95% CI: 1.4, 7.8), those who ever smoked (OR 2.7, 95% CI: 1.4, 5.1), marital status (divorce/separated) (OR 0.3, 95% CI: 0.1, 1.1), family history of diabetes (OR 2.0, 95% CI: 1.1, 3.6), and history of hypertension (OR 2.2, 95% CI: 1.2, 3.9).

All these factors were entered into a multivariable logistic regression model; the results are presented in Table 3. Having a family history of diabetes (OR 2.3, 95% CI: 1.1, 5.6 compared to no family history) and having hypertension (OR 2.3, 95% CI: 1.1, 5.2) were independently associated with a more than double odds of having diabetes.

**Table 3:** Risk factors for diabetes among respondents

Variable	Diabetes *n*=59 (10.1%)	Unadjusted OR (95% CI)	Adjusted^a^ OR (95% CI)
Population group			
Uganda rural residents	25 (16.1)	2.1 (1.1, 4.3)	1.9 (0.7, 5.3)
Uganda peri-urban residents	20 (7.6)	0.9 (0.5, 1.9)	1.2 (0.5, 3.0)
Tanzania urban teachers	14 (8.3)	1.0	1.0
Sex			
Male	22 (9.7)	1.0	
Female	37 (10.3)	1.1 (0.6, 1.9)	
Age groups			
18–29	9 (6.2)	1.0	1.0
30–39	14 (8.2)	1.4 (0.6, 3.2)	1.4 (0.4, 4.8)
40–49	13 (11.6)	2.0 (0.8, 4.8)	2.7 (0.8, 9.1)
≥ 50	17 (18.1)	3.3 (1.4, 7.8)	3.2 (0.9, 11.7)
Marital status			
Never married	40 (11.2)	1.0	1.0
Married	2 (7.1)	0.6 (0.1, 2.7)	1.3 (0.2, 6.9)
Divorced/separated	10 (22.7)	2.3 (1.1, 5.1)	0.9 (0.3, 2.7)
Widowed	2 (3.2)	0.3 (0.1, 1.1)	0.4 (0.1, 2.0)
Smoking status			
Never smoked	44 (8.6)	1.0	1.0
Ever smoked	25 (10.0)	2.7 (1.4, 5.1)	2.2 (0.9, 5.3)
Body mass index			
Normal	19 (7.5)	1.0	
Underweight	3 (17.6)	2.6 (0.7, 10.0)	
Overweight/obesity	30 (11.9)	1.7 (0.9, 3.02)	
Waist circumference			
No abdominal obesity	26 (9.2)	1.0	
Abdominal obesity	27 (10.5)	1.2 (0.7, 2.0)	
Family history of diabetes			
No	41 (8.7)	1.0	1.0
Yes	18 (15.9)	2.0 (1.1, 3.6)	2.5 (1.1, 5.6)
Hypertension			
No	25 (7.2)	1.0	1.0
Yes	25 (14.5)	2.2 (1.2, 3.9)	2.3 (1.1, 5.2)

Odds ratio of 1.0 represents the reference group. ^a^Odds ratio and 95% confidence interval adjusted for site, age groups, marital status, smoking hypertension and family history of diabetes.

Table 4 summarizes risk factors for diabetes compared to those without diabetes across the three sites. Smoking and hypertension were significant risk factors for diabetes in rural Uganda (*p*<0.01 and *p*=0.03), while abdominal obesity and a family history of diabetes were significant risk factors in peri-urban Uganda (*p*=0.04 and *p*=0.01). Age was the only risk factor associated with diabetes in Tanzania (*p*=0.03).

**Table 4:** Risk factors for diabetes by site

	Uganda rural		Uganda peri-urban		Tanzania	
			
Characteristics	Diabetes *n* (%)	*p*	Diabetes *n* (%)	*p*	Diabetes *n* (%)	*p*
Sex						
Male	9 (12.0)	0.2	11 (8.8)	0.50	2 (7.7)	1.00
Female	16 (20.0)		9 (6.6)		12 (8.4)	
Age groups						
18–29	3 (8.3)		5 (5.4)		1 (6.2)	
30–39	9 (15.8)	0.51	3 (5.9)	0.30	2 (3.2)	0.026
40–49	8 (22.9)		3 (8.6)		2 (4.8)	
≥ 50	3 (15.8)		6 (16.2)		8 (21.1)	
Smoking status						
Never smoked	12 (10.6)	0.002	18 (7.7)	1.00	14 (8.5)	1.00
Ever smoked	13 (31.0)		2 (7.1)		0 (0.0)	
Body mass index						
Normal	10 (13.2)		8 (5.5)		1 (3.2)	
Underweight	1 (25.0)	0.63	2 (15.4)	0.95		0.68
Overweight/obesity	13 (18.1)		10 (11.0)		7 (7.8)	
Waist circumference						
Normal	19 (15.4)	0.60	4 (3.5)	0.035	3 (6.5)	1.00
Abdominal obesity	6 (18.8)		16 (10.7)		5 (6.6)	
Family history of diabetes^a^						
No	22 (15.4)		10 (5.0)		9 (7.0)	
Yes	3 (25.0)	0.41	10 (16.4)	0.01	5 (12.5)	0.33
Hypertension^b^						
No	11 (10.2)		12 (6.3)	0.18	2 (4.0)	0.13
Yes	6 (27.3)	0.03	8 (11.8)		11 (13.3)	

*p* values are based on Chi square test or Fisher's exact test.

a Parents and first-degree relatives

b Systolic BP of ≥140 or diastolic BP ≥ 90 or self-report.

## Discussion

We found a high prevalence of diabetes and pre-diabetes across three sites in Tanzania and Uganda. The most dramatic finding was in rural Uganda, where the prevalence of diabetes was 16.6% and double that of our urban sites. Of concern, the vast majority of Ugandan participants with clinical diabetes were unaware of their status, in comparison to teachers in Tanzania where all participants were aware. This difference in prevalence, and the disparate knowledge of diabetes among different populations, needs both further exploration and urgent public health intervention.

Past literature has indicated that the prevalence of diabetes in rural populations is lower compared to urban populations (15). As far as we are aware, the highest reported prevalence of diabetes in a rural population in Africa was 8.8% in South Africa (16); however, our report from rural Uganda is double this figure. The prevalence of diabetes at our urban sites in Uganda and Tanzania is consistent with other studies of urban SSA populations (17).

The reasons for this high rural prevalence and lack of awareness may be due to the concentration of efforts to control NCDs in urban areas, while rural settlers may remain unaware of diabetes risk factors and prevention. However, other causes, such as changes to dietary patterns, may be important. Furthermore, the majority of participants with diabetes in our study were between 35 and 44 years old. This is consistent with other reports that diabetes affects younger people in Africa compared to western societies (1).

Substantial underdiagnosis of diabetes was evident in our study. Although all cases were captured by self-report in Tanzania, in Uganda, 65% of cases were not self-reported in peri-urban areas and 96% were not self-reported at the rural site. Studies in Uganda have reported a high proportion of undiagnosed diabetes, as high as 49%, while in rural Guinea, the proportion has been reported up to 100% (15, 18). The emphasis on the diagnosis and treatment of communicable diseases has meant that many healthcare professionals and institutions lack the tools needed to properly diagnose and treat diabetes, especially in rural settings (6). Knowledge of diabetes status may have been prominent in Tanzania due to the inclusion of primary school teachers who have relatively higher levels of education and are required to have medical examination at enrollment.

As expected, people with diabetes were more likely to have a family history of diabetes compared to people without diabetes. The high prevalence of a family history of diabetes among people without diabetes could indicate a future increase in the number of diabetes cases and is similar to other reported studies (19). Indeed, studies have found that a positive family history of diabetes is associated with a two- to six-fold increased risk of diabetes compared to those without diabetes (20). In peri-urban Uganda and Tanzania, the prevalence of a family history of diabetes among people with diabetes was similar to reports in Ghana and Ethiopia (19, 21). Although a family history of diabetes was markedly lower in rural Uganda, based on the considerable underdiagnosis of diabetes among participants themselves, it is likely due to underreporting. Participants with diabetes were more likely to have hypertension than those without diabetes, which is consistent with other studies in SSA where diabetes was associated with hypertension but not with BMI or waist circumference (22, 23). BMI and waist circumference cutoff points derived from Caucasians have not been consistent predictors of diabetes in SSA.

Managing diabetes is a challenge in many parts of the world, more so in low-income countries where shortages of medical supplies and lack of medical insurance is common. Studies conducted in hospital settings reported poor glycemic control (FBG > 7.2%) in up to 78% of patients in Tanzania (24) and 57% in Uganda (25). This information, coupled with evidence that the complications of diabetes are poorly managed in SSA, is alarming (8).

The higher proportion of undiagnosed cases of diabetes, particularly in rural communities, calls for instituting the screening of high-risk people, especially those with hypertension.

There are limitations to this study. Firstly, it is cross-sectional, and therefore the temporal direction of associations cannot be determined. The study population in Tanzania was limited to teachers and thus the findings may not be representative of the underlying urban population. Moreover, participants from Tanzania were all from urban settings, therefore limiting generalizability to rural areas. Some of the information was collected through self-report, which is subject to recall bias. The sample size was small, therefore limiting detailed analyses of risk factors. These limitations underline the importance of future large prospective studies of diabetes and other NCDs in SSA.

The findings from this study support evidence that there is a serious burden of diabetes in SSA. The prevalence in rural populations is not rare, and may be vastly under-appreciated. Large-scale, prospective cohort studies are needed to accurately quantify the burden of diabetes and its co-morbidities, understand the etiology of these diseases, and identify effective intervention and treatment strategies across diverse African populations (26). PaCT aims to scale the study to a significantly larger population that includes innovative smartphone technology for better detection and the design of interventions for this common chronic ailment. With this type of mobile technology, we will be able to receive continuous data of the behavioral patterns important for disease management, including foot care, medication compliance, and physical activity that ultimately allows for effective intervention.

## Paper context

The prevalence of diabetes is increasing in sub-Saharan Africa which is placing a great burden on health systems and society. This paper has demonstrated a high prevalence of diabetes and pre-diabetes in Tanzania and Uganda, with an alarmingly high number of undiagnosed patients in the rural community. Long term longitudinal studies are required to better quantify disease burden and measure exposure to risk factors specific for sub-Saharan Africans in order to implement appropriate local interventions.

## References

[1] International Diabetes Federation (2013). IDF diabetes atlas.

[2] World Health Organization (2016). WHO | Diabetes fact sheet.

[3] Kengne AP, Echouffo-Tcheugui JB, Sobngwi E, Mbanya JC (2013). New insights on diabetes mellitus and obesity in Africa-Part 1: prevalence, pathogenesis and comorbidities. Heart.

[4] Kengne AP, June-Rose McHiza Z, Amoah AG, Mbanya JC (2013). Cardiovascular diseases and diabetes as economic and developmental challenges in Africa. Prog Cardiovasc Dis.

[5] Perreault L, Temprosa M, Mather KJ, Horton E, Kitabchi A, Larkin M (2014). Regression from prediabetes to normal glucose regulation is associated with reduction in cardiovascular risk: results from the Diabetes Prevention Program outcomes study. Diabetes Care.

[6] Peck R, Mghamba J, Vanobberghen F, Kavishe B, Rugarabamu V, Smeeth L (2014). Preparedness of Tanzanian health facilities for outpatient primary care of hypertension and diabetes: a cross-sectional survey. Lancet Glob Health.

[7] Katende D, Mutungi G, Baisley K, Biraro S, Ikoona E, Peck R (2015). Readiness of Ugandan health services for the management of outpatients with chronic diseases. Trop Med Int Health.

[8] Dalal S, Beunza JJ, Volmink J, Adebamowo C, Bajunirwe F, Njelekela M (2011). Non-communicable diseases in sub-Saharan Africa: what we know now. Int J Epidemiol.

[9] Dalal S, Holmes MD, Laurence C, Bajunirwe F, Guwatudde D, Njelekela M (2015). Feasibility of a large cohort study in sub-Saharan Africa assessed through a four-country study. Glob Health Action.

[10] Uganda Bureau of Statistics (2014). National census main report.

[11] Higher Local Government Statistical Abstract (2007). Wakiso District.

[12] Johnston A Bushenyi District profile.

[13] International Diabetes Federation (2012). Global guideline for type 2 diabetes.

[14] World Health Organization (2006). Definition and diagnosis of diabetes mellitus and intermediate hyperlycaemia.

[15] Bahendeka S, Wesonga R, Mutungi G, Muwonge J, Neema S, Guwatudde D (2016). Prevalence and correlates of diabetes mellitus in Uganda: a population-based national survey. Trop Med Int Health.

[16] Alberts M, Urdal P, Steyn K, Stensvold I, Tverdal A, Nel JH (2005). Prevalence of cardiovascular diseases and associated risk factors in a rural black population of South Africa. Eur J Cardiovasc Prev Rehabil.

[17] Seck SM, Dia DG, Doupa D, Diop-Dia A, Thiam I, Ndong M (2015). Diabetes burden in urban and rural Senegalese populations: a cross-sectional study in 2012. Int J Endocrinol.

[18] Balde NM, Diallo I, Balde MD, Barry IS, Kaba L, Diallo MM (2007). Diabetes and impaired fasting glucose in rural and urban populations in Futa Jallon (Guinea): prevalence and associated risk factors. Diabetes Metab.

[19] Danquah I, Bedu-Addo G, Terpe KJ, Micah F, Amoako YA, Awuku YA (2012). Diabetes mellitus type 2 in urban Ghana: characteristics and associated factors. BMC Public Health.

[20] Harrison TA, Hindorff LA, Kim H, Wines RC, Bowen DJ, McGrath BB (2003). Family history of diabetes as a potential public health tool. Am J Prev Med.

[21] Abebe SM, Berhane Y, Worku A, Assefa A (2014). Diabetes mellitus in North West Ethiopia: a community based study. BMC Public Health.

[22] Alikor CA, Emem-Chioma PC (2015). Epidemiology of diabetes and impaired fasting glucose in a rural community of Nigerian Niger Delta region. Niger J Med.

[23] Fezeu L, Balkau B, Sobngwi E, Kengne AP, Vol S, Ducimetiere P (2010). Waist circumference and obesity-related abnormalities in French and Cameroonian adults: the role of urbanization and ethnicity. Int J Obes.

[24] Mwita JC, Mugusi F, Lwakatare J, Chiwanga F (2012). Hypertension control and other cardiovascular risk factors among diabetic patients at Muhimbili National Hospital, Tanzania. East Afr J Public Health.

[25] Kibirige D, Atuhe D, Sebunya R, Mwebaze R (2014). Suboptimal glycaemic and blood pressure control and screening for diabetic complications in adult ambulatory diabetic patients in Uganda: a retrospective study from a developing country. J Diabetes Metab Disord.

[26] Holmes MD, Dalal S, Volmink J, Adebamowo CA, Njelekela M, Fawzi WW (2010). Non-communicable diseases in sub-Saharan Africa: the case for cohort studies. PLoS Med.

